# The Gly82Ser mutation in *AGER* contributes to pathogenesis of pulmonary fibrosis in combined pulmonary fibrosis and emphysema (CPFE) in Japanese patients

**DOI:** 10.1038/s41598-020-69184-8

**Published:** 2020-07-30

**Authors:** Takumi Kinjo, Yoshiaki Kitaguchi, Yunden Droma, Masanori Yasuo, Yosuke Wada, Fumika Ueno, Masao Ota, Masayuki Hanaoka

**Affiliations:** 1 First Department of Internal Medicine, Shinshu University School of Medicine, Matsumoto, Japan; 2 Division of Hepatology and Gastroenterology, Department of Medicine, Shinshu University School of Medicine, Matsumoto, Japan

**Keywords:** Genetics, Diseases, Pathogenesis

## Abstract

The dominant pathogenesis underlying the combined pulmonary fibrosis and emphysema (CPFE) remains unresolved. The receptor for advanced glycation end-products (RAGE) is highly expressed in lung tissues and interacts with distinct multiple ligands, implicating it in certain lung diseases. To elucidate the pathogenesis of CPFE, we genotyped three single nucleotide polymorphisms (SNPs: rs2070600, rs1800625, and rs2853807) of the gene encoding RAGE (*AGER*) in 111 CPFE patients and 337 chronic obstructive pulmonary disease (COPD) patients of Japanese by using StepOne Real-Time PCR System for SNP genotyping assay. Serum levels of soluble RAGE (sRAGE) were measured by ELISA. We found that the allele frequency of rs2070600 was significantly different between the two groups [corrected P (Pc) = 0.015]. In addition, the minor allele was associated with CPFE patients relative to COPD patients in a dominant effect model (Odds Ratio = 1.93; Pc = 0.018). Moreover, the serum sRAGE level was significantly lower in the CPFE group than the COPD group (P = 0.014). The rs2070600 minor allele was significantly associated with reduced sRAGE level in CPFE patients and independently affected sRAGE level reduction in this group (P = 0.020). We concluded that the *AGER* rs2070600 minor allele (Gly82Ser mutation) is associated with the pathogenesis of pulmonary fibrosis in CPFE in Japanese patients.

## Introduction

Some patients with chronic obstructive pulmonary disease (COPD) have concomitant pulmonary fibrosis in addition to emphysema, which is known as combined pulmonary fibrosis and emphysema (CPFE)^1^. CPFE is characterized by upper-lobe emphysema and lower-lobe fibrosis on high-resolution computed tomography (HRCT) of the chest and preserved lung volume with serious diminished gas exchange capacity in pulmonary function tests. Because of frequent complications with pulmonary hypertension, acute lung injury, and lung cancer, the prognosis of CPFE is significantly poor relative to that of patients with emphysema only^1,2^. To date, the pathogenesis of CPFE remains to be elucidated in terms of how the two conditions co-occur when they are considered to have different physiological and radiological characteristics. Our previous study demonstrated that enrichment of gene expression in fibrotic tissue differs from that in emphysematous lesions in lung tissues from patients with CPFE^3^. Genetic factors have been suggested to be involved in CPFE pathogenesis.

The receptor for advanced glycation end-products (RAGE) is a multiligand member of the immunoglobulin superfamily of cell surface molecules^4^. It interacts with distinct multiple ligands in ways that implicate it in homeostasis, development, inflammation, and certain lung diseases through modulation of multiple intracellular signaling pathways^4^. RAGE is constitutively highly expressed in the lung^5^, where decreased expression is associated with pulmonary fibrosis^6^ and lung cancer^7^ and upregulated expression has been observed in COPD^8^. The soluble form of RAGE (sRAGE) in the circulation is produced through alternative splicing of RAGE pre-mRNA or proteolytic cleavage of full-length RAGE^9^. The sRAGE protein binds ligands of RAGE and can prevent the adverse effects of RAGE signaling. The level of sRAGE may serve as a proxy for the biological function of RAGE^10^.

The gene encoding RAGE (*AGER*) lies on chromosome 6 and comprises 11 exons interlaced by 10 introns^11^. The single nucleotide polymorphism (SNP) rs2070600 (Gly82Ser) on exon 3 is located at the ligand-binding site and works at the N-linked glycosylation site of the protein^11,12^. This SNP has been demonstrated to be significantly associated with sRAGE level^13^, lung function^14,15^, COPD^16,17^, and idiopathic pulmonary fibrosis (IPF)^18^. The SNP rs1800625 is located at the gene promoter and functions to increase RAGE expression and sRAGE levels^12^. This SNP is involved in the pathogenesis of inflammatory diseases and the severity of cystic fibrosis^19^. In addition, SNP rs2853807 is located in intron 8 of *AGER* and has been evaluated for an association with IPF in Japanese patients^18^.

We hypothesized that these SNPs in *AGER* could be involved in the pathogenetic differences between CPFE and COPD. In the current study, we genotyped rs2070600, rs1800625, and rs2853807 of *AGER* in Japanese patients with CPFE and COPD and investigated the associations of these SNPs with CPFE relative to COPD. We also assessed associations of the three SNPs with serum sRAGE levels in these patients.

## Results

### CPFE and COPD patients

A total of 111 patients with CPFE and 337 patients with COPD were included. These groups did not differ significantly for sex ratio, average age, or smoking history (P = 0.57, 0.57, and 0.41, respectively; Table 1).

**Table 1:** Clinical characteristics of the patients with CPFE and COPD.

Parameters	CPFE	COPD	P
Number	111	337	
Sex ratio (female/male)	3/108	13/324	0.57*
Age (years)	72.4 ± 7.0	71.9 ± 7.0	0.57**
BMI (kg/m^2^)	22.7 ± 2.96	21.9 ± 3.23	0.02**
Smoking history (pack-years)	53.8 ± 25.1	56.4 ± 29.0	0.41**
Lung cancer (+ / − , n) (%)	82/29 (73.9)	146/191 (43.3)	< 0.0001*
Pulmonary function tests			
FVC (% predicted)	96.7 (84.8–109.5)	95.9 (82.1–111.6)	0.91***
FEV_1_ (% predicted)	82.1 (70.0–93.2)	68.5 (51.1–81.4)	< 0.0001***
FEV_1_/FVC (%)	68.5 (60.5–75.1)	58.3 (47.0–63.8)	< 0.0001***
RV (% predicted)	118.5 (95.2–146.2)	143.0 (126.4–168.2)	< 0.0001***
TLC (% predicted)	107.3 (93.2–116.8)	113.9 (104.0–126.2)	< 0.0001***
DLco (% predicted)	49.7 (39.5–61.6)	62.9 (46.1–77.8)	< 0.0001***
Chest HRCT shadows for emphysema			
LAA score	8 (5–11)	7 (4–12)	0.08***
Chest HRCT shadows for fibrosis			
Extent of interstitial change, n (%)			
Minimal	49 (44.1)	NF	
Moderate	39 (35.2)		
Severe	23 (20.7)		
Radiological patterns of interstitial changes, n (%)			
Honeycombing	67 (60.4)	NF	
Reticular opacity	83 (74.8)		
Ground glass opacity	56 (50.5)		
Traction bronchiectasis	27 (24.3)		
Consolidation	0 (0)		
Treatment for COPD (inhaled corticosteroids and bronchodilators)			
LAMA	5	36	
LABA	6	9	
ICS	1	1	
LAMA + LABA	5	11	
LAMA + ICS	0	2	
LABA + ICS	4	9	
LAMA + LABA + ICS	2	9	
No pharmacotherapy	88	260	
Treatment for pulmonary fibrosis (anti-fibrotic agents, systemic steroids and immunosuppressants)			
Anti-fibrotic agents			
Nintedanib	2	NA	
Pirfenidone	1		
No pharmacotherapy	108		
Systemic steroids	0		
Immunosuppressants	0		

Date are expressed as mean ± standard deviation (SD) or median (interquartile range, IQR) unless otherwise stated.

CPFE, combined pulmonary fibrosis and emphysema; COPD, chronic obstructive pulmonary disease; BMI, body mass index; FVC, forced vital capacity; FEV_1_, forced expiratory volume in 1 s; RV, residual volume; TLC, total lung capacity; DLco, diffusing capacity of lung for carbon monoxide; LAA, low attenuation areas; LAMA, long-acting muscarinic antagonist (Tiotropium, Glycopyrronium, Aclidinium, Umeclidinium); LABA, long-acting beta_2_-agonist (Indacaterol, Salmeterol, Vilanterol, Formoterol, Olodaterol); ICS, inhaled corticosteroid (Fluticasone, Budesonide, Ciclesonide, Beclometasone); NF, not found; NA, not applicable.

P values were analyzed by 2 × 2 contingency table with*, unpaired *t*-test with** and Mann–Whitney U test with***.

Pulmonary function tests strictly divided the patients into CPFE and COPD groups on pathophysiological impairment (Table 1). The COPD patients showed airflow limitation with reductions in the predicted percentage of forced expiratory volume in 1 s (%FEV_1_) and the ratio of FEV_1_ to forced vital capacity (FVC). The CPFE patients presented with reductions in the predicted percentage of diffusing capacity of lung for carbon monoxide (%DLco). The predicted percentages of residual volume (%RV) and total lung capacity (%TLC) were relatively preserved in the CPFE patients (Table 1).

The chest HRCT was performed in all patients including both COPD and CPFE groups. The low attenuation area (LAA) in the bilateral upper, middle, and lower lung fields was scored to evaluate the extent of emphysema in both groups. The LAA scores did not differ significantly between the CPFE and COPD groups (Table 1). Regarding the extent of fibrosis on chest HRCT, it was minimal in 44.1% of patients, moderate in 35.1%, and severe in 20.7%, using a scale of severity for patients with CPFE (Table 1). The distributions of honeycombing, reticular opacity, ground glass opacity, traction bronchiectasis and consolidation on HRCT were 60.4%, 74.8%, 50.5%, 24.3% and 0% in patients with CPFE, respectively (Table 1). The interstitial changes were absence in patients with COPD on HRCT images (Table 1).

The inhaled corticosteroids were administrated to 7 patients of the CPFE group and 21 patients of the COPD group (Table 1). There is no history of acute exacerbation at the time of diagnosis in the patients with CPFE, therefore, no patients were treated with systemic steroids or immunosuppressants in the CPFE group. Three patients with CPFE were treated with anti-fibrotic agents (Table 1).

### Significant association of rs2070600 with CPFE relative to COPD

The genotype distributions of the rs2070600, rs1800625, and rs2853807 all were in Hardy–Weinberg equilibrium in both patient groups. The rs2070600 showed significant differences in genotype distribution between CPFE and COPD patients (Table 2). CPFE patients had a significantly higher minor allele frequency of rs2070600 compared to COPD patients (0.171 vs 0.101; *Pc* = 0.015; OR = 1.84, 95% CI = 1.20–2.83; Table 2). In addition, the minor allele of the rs2070600 was linked to CPFE patients relative to the COPD patients in the dominant effect model (OR = 1.93, 95% CI = 1.20–3.12; *Pc* = 0.018; Table 2). The rs1800625 and rs2853807 SNPs showed no significant differences in genotype distributions or allele frequencies between the two patient groups (Table S1).

**Table 2:** Genotype distribution and allele frequency of the rs2070600 SNP between the CPFE and COPD groups.

	Genotype/Allele	CPFE (n = 111)	COPD (n = 337)	P*	Pc	OR (95% CI)
**SNP**	rs2070600 (C > T)					
Genotype	TT/CT/CC (n)	2/34/75	1/66/270	0.011	0.033	
	TT/CT/CC (freq)	0.018/0.306/0.676	0.003/0.196/0.801			
Allele	T/C (n)	38/184	68/606	0.005	0.015	1.84 (1.20–2.83)
	T/C (freq)	0.171/0.829	0.101/0.899			
Dominant model	TT + CT/CC (freq)	0.324/0.676	0.199/0.801	0.006	0.018	1.93 (1.20–3.12)
Recessive model	TT/CT + CC (freq)	0.018/0.982	0.003/0.997	0.092	0.276	6.17 (0.55–68.66)

CPFE, combined pulmonary fibrosis and emphysema; COPD, chronic obstructive pulmonary disease; SNP, single nucleotide polymorphism; n, number; freq, frequency; Pc, corrected P value; OR, odds ratio; CI, confidence interval.

*By Chi-square test with 2 × 3 contingency table; otherwise, by Chi-square test with 2 × 2 contingency table for allele, dominant model, and recessive model. Supposing the minor allele (m) and major allele (M), the dominant model compares mm + mM versus MM; and the recessive model compares mm versus mM + MM. If the number was less than 5, Fisher’s exact test was applied instead.

### Serum sRAGE levels

**Figure 1:**
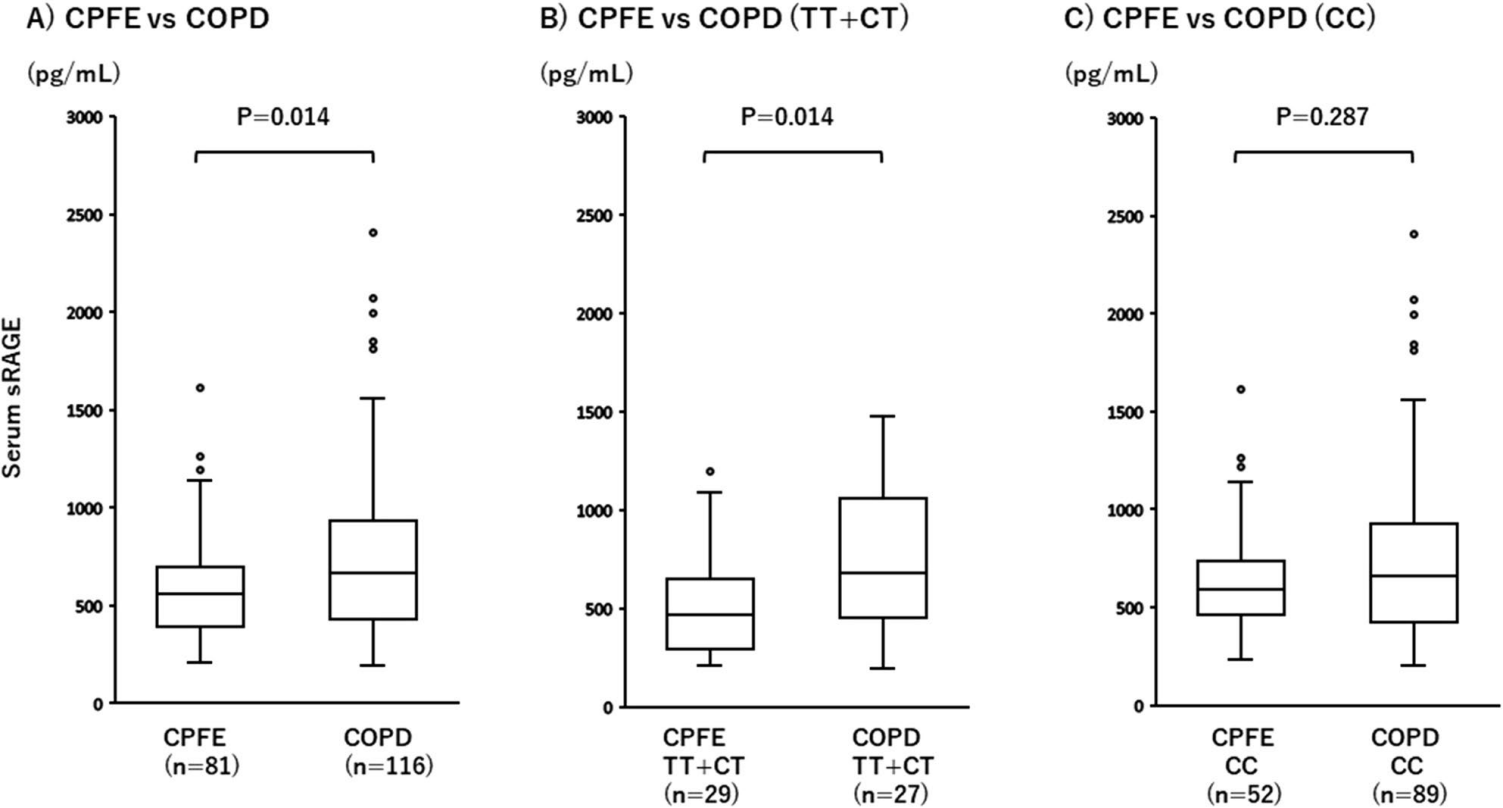
Comparisons of serum sRAGE levels between the CPFE and COPD groups. (**A**) Serum sRAGE level was significantly lower in CPFE compared to COPD patients (P = 0.014). (**B**) Serum sRAGE level was significantly lower in CPFE compared to COPD patients carrying the rs2070600 minor allele (P = 0.014). (**C**) Serum sRAGE levels did not differ between the CPFE and COPD patients without the rs2070600 minor allele (P = 0.287). T: rs2070600 minor allele; C: rs2070600 major allele.

**Figure 2:**
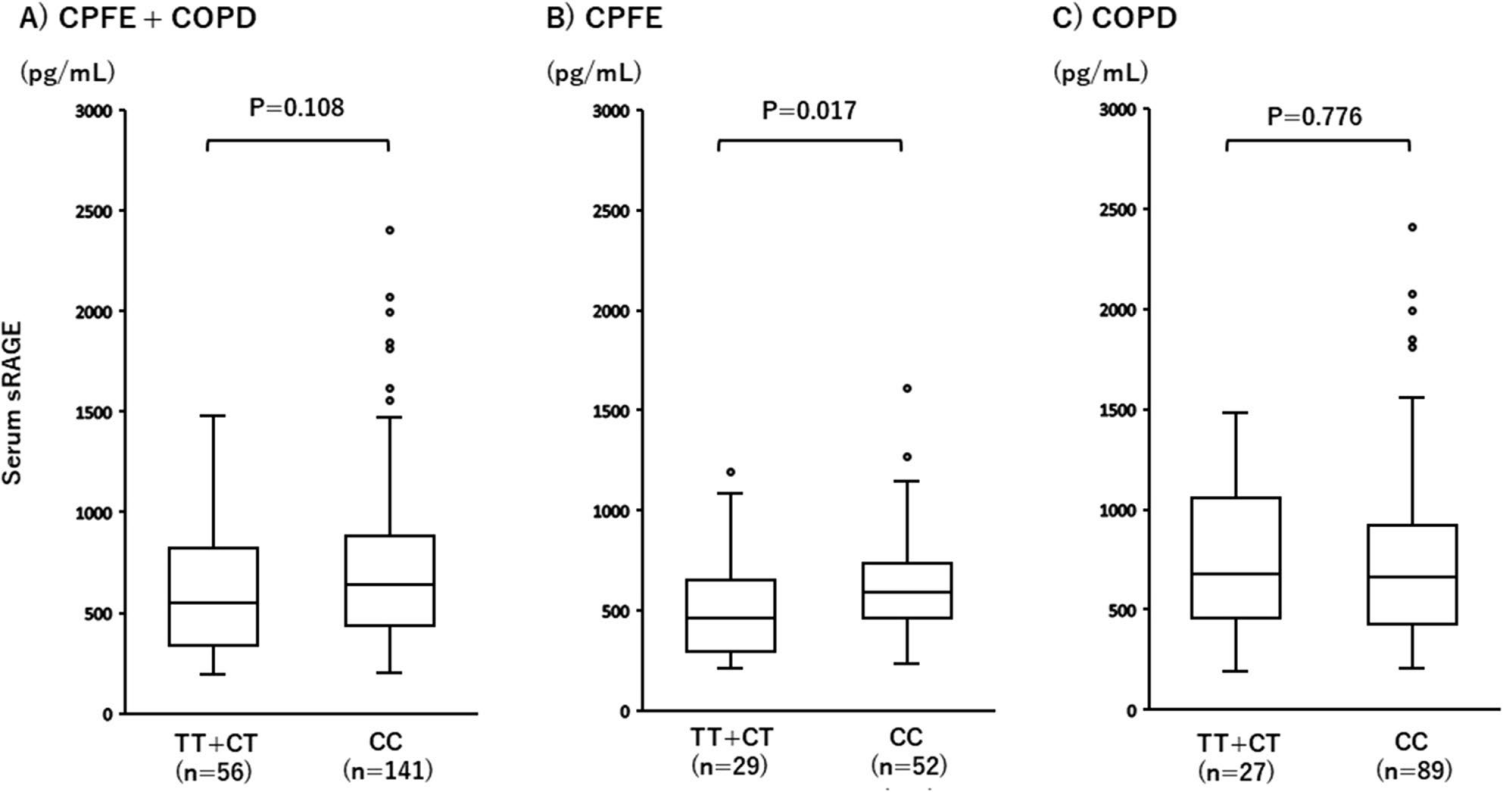
Comparisons of serum sRAGE levels between participants with and without the rs2070600 minor allele. (**A**) Serum sRAGE levels did not differ significantly between the total patients of CPFE and COPD with and without the rs2070600 minor allele (P = 0.108). (**B**) Serum sRAGE levels were significantly lower in CPFE patients carrying the rs2070600 minor allele compared to noncarriers (P = 0.017). (**C**) Serum sRAGE levels did not differ between COPD patients with and without the rs2070600 minor allele (P = 0.776). T: rs2070600 minor allele; C: rs2070600 major allele.

Serum sRAGE levels were measured in 81 male CPFE patients and 116 male COPD patients. The levels were significantly lower in the CPFE patients than the COPD patients (598.6 ± 286.5 pg/ml vs. 754.9 ± 435.7 pg/ml, P = 0.014; Fig. 1A), and moreover, significantly lower in the CPFE patients than the COPD patients carrying the rs2070600 minor allele (503.8 ± 249.7 pg/ml vs. 743.4 ± 365.3 pg/ml, P = 0.014; Fig. 1B). However, the serum sRAGE levels did not differ between the CPFE and COPD patients without the rs2070600 minor allele (651.5 ± 294.1 pg/ml vs. 758.4 ± 456.7 pg/ml, P = 0.287; Fig. 1C). On the other hand, there was no significant difference of the sRAGE levels between the whole patients (CPFE and COPD) with and without the rs2070600 minor allele (619.3 ± 330.8 pg/ml vs. 719.0 ± 406.6 pg/ml, P = 0.108; Fig. 2A), while there was indeed a significant difference of the serum sRAGE levels between the CPFE patients with and without the rs2070600 minor allele (503.8 ± 249.7 pg/ml vs. 651.5 ± 294.1 pg/ml, P = 0.017; Fig. 2B). However, the serum sRAGE levels did not differ between the COPD patients with and without the rs2070600 minor allele (743.4 ± 365.3 pg/ml vs. 758.4 ± 456.7 pg/ml, P = 0.776; Fig. 2C). Taking the results together, it is suggested that the rs2070600 minor allele is significantly associated with the serum sRAGE level in the CPFE group. Moreover, among the clinical and genetic variables evaluated in the present study, multivariate linear regression analysis showed an independent correlation of the rs2070600 minor allele with the reduced sRAGE levels in the CPFE patients (P = 0.02, t = -2.34, β = -0.32; Table 3). In contrast, the serum sRAGE levels were not significantly associated with the rs2070600 in COPD patients (Fig. 2C, Table 4).

**Table 3:** Correlation between serum sRAGE levels and clinical variates in CPFE.

Variables	t	β	P*
Univariate analysis			
Age (years)	2.59	0.32	0.01
BMI (kg/m^2^)	0.15	0.02	0.88
Smoking history (pack-years)	− 0.20	− 0.03	0.84
FVC (% predicted)	0.57	0.07	0.57
FEV_1_ (% predicted)	1.24	0.16	0.22
DLco (% predicted)	1.48	0.19	0.14
Lung cancer	− 0.29	− 0.04	0.77
rs2070600 minor allele (+ /−)	− 2.51	− 0.31	0.015
rs1800625 minor allele (+ /−)	0.09	0.01	0.93
rs2853807 minor allele (+ /−)	− 0.88	− 0.11	0.38

	t	β	P**
Multivariate analysis			
Age (years)	1.88	0.24	0.07
BMI (kg/m^2^)	− 0.01	− 0.001	0.99
Smoking history (pack-years)	0.76	0.10	0.45
FVC (% predicted)	− 1.16	− 0.24	0.25
FEV_1_ (% predicted)	1.43	0.30	0.16
DLco (% predicted)	1.36	0.19	0.18
Lung cancer	− 0.92	− 0.12	0.36
rs2070600 minor allele (+ /−)	− 2.34	− 0.32	0.02
rs1800625 minor allele (+ /−)	− 0.59	− 0.08	0.56
rs2853807 minor allele (+ /− )	− 0.76	− 0.10	0.45

sRAGE, soluble receptor for advanced glycation end product; CPFE, combined pulmonary fibrosis and emphysema; BMI, body mass index; FVC, forced vital capacity; FEV_1_, forced expiratory volume in 1 s; DL_CO_, diffusing capacity of lung for carbon monoxide.

*Univariate linear regression analysis.

**Multivariate linear regression analysis.

**Table 4:** Correlation between serum sRAGE levels and clinical variates in COPD.

Variables	t	β	P*
Univariate analysis			
Age (years)	1.93	0.18	0.06
BMI (kg/m^2^)	0.37	0.04	0.71
Smoking history (pack-years)	− 2.06	− 0.19	0.04
FVC (% predicted)	0.10	0.01	0.92
FEV_1_ (% predicted)	0.53	0.05	0.60
DLco (% predicted)	0.38	0.04	0.70
Lung cancer	− 2.63	− 0.24	0.01
rs2070600 minor allele (+ /−)	− 0.57	− 0.05	0.57
rs1800625 minor allele (+ /−)	0.50	0.05	0.62
rs2853807 minor allele (+ /−)	1.72	0.16	0.09

	t	β	P**
Multivariate analysis			
Age (years)	1.86	0.18	0.07
BMI (kg/m^2^)	0.06	0.007	0.95
Smoking history (pack-years)	− 1.70	− 0.17	0.09
FVC (% predicted)	− 0.58	− 0.10	0.56
FEV_1_ (% predicted)	0.75	0.13	0.46
DLco (% predicted)	0.22	0.02	0.82
Lung cancer	− 2.32	− 0.22	0.02
rs2070600 minor allele (+ /−)	− 0.12	− 0.01	0.91
rs1800625 minor allele (+ /−)	0.70	0.07	0.49
rs2853807 minor allele (+ /−)	1.89	0.18	0.06

sRAGE, soluble receptor for advanced glycation end product; CPFE, combined pulmonary fibrosis and emphysema; BMI, body mass index; FVC, forced vital capacity; FEV_1_, forced expiratory volume in 1 s; DL_CO_, diffusing capacity of lung for carbon monoxide.

*Univariate linear regression analysis.

**Multivariate linear regression analysis.

The remaining two SNPs, rs1800625 and rs2853807, showed no significant associations with the serum sRAGE level in either patient group.

### No significant associations of the three SNPs with lung cancer in the CPFE patients

Lung cancer is a well-known frequent complication of CPFE^20^. In recruitment of subjects for this study, we did not exclude patients with lung cancer, which was present in 73.9% of the CPFE group and 43.3% of COPD group (P < 0.0001; Table 1). Among the total of 448 patients of both CPFE and COPD, 228 patients were complicated with lung cancer. Genotype distributions and allele frequencies of rs2070600, rs1800625, and rs2853807 did not differ significantly between patients with and without lung cancer in the total group (Table S2). Although serum sRAGE levels were significantly lower among the total patients of CPFE and COPD with lung cancer than those without (612.6 ± 319.9 pg/ml vs. 826.2 ± 456.4 pg/ml, P = 0.0007; Figure S1A), they did not differ between those with and without lung cancer in the CPFE group (586.4 ± 280.4 pg/ml vs. 656.8 ± 318.5 pg/ml, P = 0.406; Figure S1B). Overall, the findings suggested that the SNPs rs2070600, rs1800625, and rs2853807 were not significantly associated with lung cancer in the present CPFE group.

## Discussion

The remarkable finding of the present study was that the rs2070600 SNP of *AGER* was significantly associated with susceptibility to CPFE relative to COPD. The CPFE patients carrying the minor allele of rs2070600 showed significantly reduced serum sRAGE compared to COPD patients. In addition, the serum sRAGE was significantly lower with versus without the rs2070600 minor allele in CPFE patients, and this minor allele independently correlated with the reduced sRAGE levels in this group. These results suggest that a down-regulated RAGE pathway resulting from the mutation of Gly82Ser (rs2070600) in *AGER* is likely involved in the pathogenesis of pulmonary fibrosis in CPFE.

The presence of lung cancer at a significantly higher prevalence in patients with CPFE compared to those with COPD leaves open the potential for confounding effect if lung cancer is associated with the *AGER* genetic variations. However, a potential link is currently controversial, precluding firm conclusions. Two studies in the Chinese population found a significant association of rs2070600 with lung cancer^21,22^, but studies in Caucasian^23^ and Japanese^24^ patients did not. Meanwhile, Wang et al. could not replicate the significant association in another study in Chinese patients^25^. In the current work, we also found no associations of rs2070600, rs1800625, or rs2853807 with lung cancer in CPFE or COPD patients (Table S2).

The current results do suggest an effect of rs2070600 on CPFE susceptibility relative to COPD, based on patterns of genotype distribution, allele frequency, and the dominant model of the minor allele. Yamaguchi et al.^18^ reported a significant association of rs2070600 with IPF among Japanese patients in a dominant model. In contrast, Manichaikul et al.^26^ found no such association in a population of Caucasian patients with IPF, but did find that the rs2070600 minor allele was significantly associated with reduced sRAGE levels in these patients. The association of rs2070600 with COPD also is controversial. One genome-wide association study found a significant association with an emphysema-related phenotype in Caucasian patients and with reduced serum sRAGE levels^16^. While Young et al. suggested that the minor allele of rs2070600 was associated with protection against COPD in healthy Caucasian smokers^17^. There is no available information about the associations of *AGER* variants with COPD in Japanese population at present. The LAA score did not differ significantly between CPFE and COPD patients in the current study (Table 1), so the present result, that the significant association of rs2070600 SNP of *AGER* with CPFE, likely depended on the presence of fibrosis. The rs2070600 minor allele is proposed to be associated with the pathogenesis of pulmonary fibrosis in CPFE.

Whether emphysema and fibrotic lesions progress independently or influence each other in CPFE remains unclear. A distinct radiological feature of CPFE is the presence of large, thick-walled cystic lesions^27^. These thick-walled large cysts probably represent the development of pulmonary fibrosis within the emphysematous lung, and seem to be enlarged due to retraction forces in the fibrotic lesions^28^. Katzenstein et al. reported that more than half of lobectomy specimens excised from smokers with lung cancer had interstitial fibrosis pathologically; however, these patients had no clinical evidence of interstitial lung disease radiologically, and in some of them, emphysema was the only CT finding^29^. These results suggested that CPFE may arise as a development of fibrosis superimposed on a known history of emphysema. Indeed, previous studies have found that pulmonary fibrosis occurs subsequent to pulmonary emphysema^29–31^. Based on these reports and present results, we suggest that pulmonary fibrosis may occur subsequent to pulmonary emphysema among patients carrying the *AGER* rs2070600 minor allele (Gly82Ser mutation).

RAGE contains an extracellular domain, a single transmembrane-spanning domain, and a 43-amino acid cytosolic tail. As noted, the sRAGE is produced by either alternative splicing events of the mRNA of *AGER* or proteolytic cleavage of membrane-bound RAGE and secreted by cells. The sRAGE proteins in circulation bind to the ligands of RAGE and can inhibit the adverse effects of RAGE signaling^4,11^. Lung cancer^25^, emphysema^16,32^, and IPF^10,18,26^ are associated with significantly decreased serum sRAGE levels compared with healthy controls. In the present study, the rs2070600 minor allele showed a significant association with reduced serum sRAGE levels in CPFE patients (Fig. 2B) but not in COPD patients (Fig. 2C). In addition, this reduced serum sRAGE in CPFE patients was not affected by lung cancer (Figure S1B). These results demonstrated that the presence of the rs2070600 minor allele independently affected serum sRAGE level reductions in the CPFE patients (Table 3).

The rs2070600 is a missense variation that results in the substitution of serine for glycine at codon 82 (Gly82Ser) in the RAGE protein. The mutation is located in exon 3, a putative site of the ligand-binding V domain of *AGER*, and modifies RAGE ligand-binding structure and affinity for ligands.^11,12^ Taking the evidence together, rs2070600 seems to alter RAGE function, leading to reduced serum sRAGE in CPFE, confirming a genetic role in the pathogenesis of pulmonary fibrosis in CPFE. We speculate that the rs2070600 minor allele of the *AGER* might regulate the down expression of RAGE in lung tissue and circulation, resulting in improper cellular adhesion^33^, differentiation, and repair mechanisms^34^, leading to matrix deposition^35^ and impaired epithelial regeneration^36^ in the pathogenesis of fibrosis. Further studies on *AGER* mRNA expression in lung tissue/circulation of CPFE patients will help elucidate the signaling pathway involving RAGE and its role in CPFE pathogenesis.

CPFE was diagnosed radiologically whereas the COPD was diagnosed based on both symptomatic and physiological impairment^1,2,39^. The notice of the present study was that all of the patients were performed HRCT and pulmonary function tests for the purposes of diagnosis and distinctness of phenotypes of CPFE and COPD on HRCT. In addition to the pulmonary function tests in consistent with the characteristics of CPFE and COPD correspondingly^20^, the HRCT shadows definitely exhibited absence of interstitial changes in COPD patients, in spite of the emphysema in both CPFE and COPD groups (Table 1). Thus, the phenotypes of CPFE and COPD were strictly divided in HRCT images based on presence or absence of pulmonary fibrosis for detection of the *AGER* genetic association with pulmonary fibrosis in CPFE relative to COPD. The results suggested that the patients with emphysema carrying the *AGER* rs2070600 SNP was susceptible to development of CPFE.

The present study has several limitations. The major limitation is the prevalence of lung cancer among participants. Theoretically, it would have been ideal to exclude patients with lung cancer in the study subjects. However, this comorbidity is common in patients with CPFE, and excluding participants with lung cancer would have yielded insufficient sample sizes. Nevertheless, the supplemental statistical analysis showed no associations of the three SNPs with lung cancer (Table S2) or of the lung cancer with reduced sRAGE levels in CPFE group (Figure S1B). Thus, lung cancer was not likely a confounding factor in the present study. A future study with larger sample sizes is expected to confirm this issue. Additional limitation is the small sample size and narrow focus involving few SNPs of the gene. Therefore, the possibility of type I error cannot be excluded. However, the power calculations based on study subjects of 111 CPFE patients and 337 COPD patients demonstrated sufficient detection power (0.89) at the 0.05 level of significance for rs2070600. The third limitation is lack of a follow-up of observation to verify the susceptibility to development of pulmonary fibrosis in patients with pulmonary emphysema who carry the rs2070600 minor allele. Lastly, the sRAGE levels were measured in 81 out of 111 patients with CPFE and 116 out of 337 patients with COPD, because the serum samples were available only from 81 patients with CPFE and 116 patients with COPD. Nevertheless, the result showed that the serum sRAGE level was significantly lower in the CPFE group than the COPD group (p = 0.014).

In conclusion, the *AGER* rs2070600 SNP (Gly82Ser mutation) was associated with the pathogenesis of pulmonary fibrosis in CPFE in Japanese patients.

## Patients and methods

### Patients

The Ethics Committee of Shinshu University approved this study (permission number 619). The study protocols were performed in accordance with the principles outlined in the Declaration of Helsinki of the World Medical Association.

The patients in the present study were consecutive patients with CPFE or COPD at the first medical consultation in our institute (Shinshu University Hospital, Matsumoto, Japan) during a period from December 2006 to March 2019. All were Japanese. The CPFE diagnosis was based on the simultaneous presence of emphysema predominantly in the upper lung fields and diffuse pulmonary fibrosis mostly in the lower lung fields on chest HRCT^1,2^. The extent of emphysema and interstitial change was evaluated semi-quantitatively on the chest HRCT using methods described previously^30,37,38^. COPD diagnosis was based on smoking history, clinical symptoms, and pulmonary function tests according to the Global Initiative for Chronic Obstructive Lung Disease (GOLD) Report^39^. In addition, chest HRCT was performed in patients with COPD to confirm the presence of emphysema and absence of pulmonary fibrosis for distinction COPD from CPFE on lung radiology. The blood samples were collected from the patients at the time of diagnosis of CPFE or COPD after obtaining the written informed consent from the patients. The status of CPFE and COPD was stable with no signs of respiratory tract infections and without exacerbation in three months preceding the study.

Patients with CPFE and COPD all had a history of smoking more than 10 pack-years. The complication of lung cancer was included in the present study because it is a clinical feature of CPFE^20^. Basically, CPFE or COPD were classified in category of complication with lung cancer and without lung cancer at the time of diagnosis. The lung cancer developed during the follow-up/treatment period of CPFE or COPD were classified in category of complication without lung cancer.

Patients with bronchiectasis, asthma, chronic hypersensitivity pneumonitis, interstitial lung disease due to autoimmune disease, drug-induced lung disease, sarcoidosis, pneumoconiosis, late sequelae of pulmonary tuberculosis, or chronic pulmonary infections such as aspergillosis and nontuberculous mycobacterial disease were excluded from the study.

### Pulmonary function tests

All the patients underwent pulmonary function tests including spirometry and measurements of the diffusion capacity of the lung for carbon monoxide (DLco), residual volume (RV), and total lung capacity (TLC) by using CHESTAC-8900 (CHEST Co., Ltd., Tokyo, Japan).

### Chest HRCT and image criteria

The chest HRCT was taken by using a multi-detector CT scanner (LightSpeed VCT, GE Healthcare, Little Chalfont, Buckinghamshire, UK) at inspiratory breath-holding status in supine position for all patients in CPFE and COPD groups. The extent of emphysema was scored visually based on the identification of low attenuation area (LAA) in the bilateral upper, middle, and lower lung fields according to the methods of Goddard et al.^38^ The LAA score was calculated by summing scores of six lung fields. The extent of interstitial change was scored visually to grade the severity as minimum, moderate, and severe, as previously described^37^. The radiological patterns of interstitial changes on HRCT were honeycombing, reticular opacity, ground glass opacity, traction bronchiectasis and consolidation according to the category of previous description^30^.

The chest HRCT images were reviewed by two expert pulmonologists (Y.K. and T.K., with 21 and 10 years of experience, respectively) in a model of blind to clinical information of patients. They separately scored the extent of emphysema (the LAA score) and graded the severity on extent of interstitial change. With the cases of disagreement, discussion and re-evaluation were performed to reach the agreement.

### Genotyping of SNPs

The genomic DNA samples were extracted from the venous blood of all patients using QuickGene800 (FUJI FILM, Tokyo, Japan). Allele discrimination was performed for the rs2070600, rs1800625, and rs2853807 SNPs of the *AGER* with the StepOne Real-Time PCR System (Thermo Fisher Scientific, Waltham, MA, USA) following the manufacturer’s instructions. After thermal cycling, genotype data were automatically acquired and analyzed using sequence detection software (StepOne Software v2.3, Thermo Fisher Scientific).

### Measurement of serum sRAGE

Serum samples were obtained from patients and stored at -80℃ until measurement. We measured serum sRAGE level using a commercially available ELISA kit (Quantikine; R&D Systems, Minneapolis, MN, USA) according to the manufacturer’s protocol. The measurements were performed in duplicate. The intra-assay and inter-assay coefficients of variations were 4.8% and 6.6%, respectively.

### Statistical analyses

For the data in normal distribution, the continuous variables are expressed as mean ± standard deviation (SD) and the differences between two groups were analyzed by the Student’s t-test. On the other hand, for the data with skewed distribution, the continuous variables are expressed as median with interquartile range (IQR) and the differences between the two groups were analyzed by the Mann–Whitney U test. The differences in categorical data were analyzed using 2 × 2 contingency tables. For each SNP in the CPFE and COPD patients, we calculated the Hardy–Weinberg equilibrium individually using the Genepop software package^40^. Differences in genetic information between CPFE and COPD patients were analyzed by Chi square tests. The strengths of the minor allele in the CPFE patients were estimated by odds ratios (ORs) with 95% confidence intervals (CIs). Power analyses were performed using G*Power version 3.1.9.6^41^. P values were corrected (Pc) for multiple hypothesis tests using Bonferroni’s method^42^. A multivariate linear regression analysis was conducted to investigate the independent effects of the relevant factors on serum sRAGE levels. P and Pc values of less than 0.05 were considered significant.

### Data availability

The datasets generated and/or analysed during the current study are available from the corresponding author on reasonable request.

## Supplementary information

Supplementary information 1.

Supplementary information 2.
