# Supplementary material for: The Gly82Ser mutation in AGER contributes to pathogenesis of pulmonary fibrosis in combined pulmonary fibrosis and emphysema (CPFE) in Japanese patients
Source: Sci Rep. 2020 Jul 30;10:12811. doi: 10.1038/s41598-020-69184-8 (PMC7393115; doi:10.1038/s41598-020-69184-8)
Supplement: Supplementary file 1 — Supplementary information 1. [file 41598_2020_69184_MOESM1_ESM.docx]

**Online supplements**

**The Gly82Ser mutation in *AGER* contributes to pathogenesis of pulmonary fibrosis in combined pulmonary fibrosis and emphysema (CPFE) in Japanese patients**

Takumi Kinjo, MD^1^; Yoshiaki Kitaguchi, MD, PhD^1^*; Yunden Droma, MD, PhD^1^; Masanori Yasuo, MD, PhD^1^; Yosuke Wada, MD, PhD^1^; Fumika Ueno, MD^1^; Masao Ota, PhD^2^; Masayuki Hanaoka, MD, PhD^1^

^1^First Department of Internal Medicine, Shinshu University School of Medicine, Matsumoto, Japan.

^2^Department of Medicine, Division of Hepatology and Gastroenterology, Shinshu University School of Medicine, Matsumoto, Japan

* corresponding author:

E-mail address: [kitaguti@shinshu-u.ac.jp](about:blank)

**Figure Legend**

**Figure S1. Comparison of serum sRAGE levels between patients with and without lung cancer.** A: Serum sRAGE level was significantly lower in the patients (CPFE + COPD) with lung cancer compared to those without lung cancer (P = 0.0007). B: Serum sRAGE levels did not differ between CPFE patients with and without lung cancer (P = 0.406). C: Serum sRAGE level was significantly lower in COPD patients with lung cancer compared to those without lung cancer (P = 0.005).

**Table S1. Genotype distribution and allele frequency of the rs1800625 and rs2853807 SNPs between the CPFE and COPD groups**

|  | **Genotype/Allele** | CPFE  n=111 | COPD  n=337 | P * | Pc | OR (95%CI) |
| --- | --- | --- | --- | --- | --- | --- |
| **SNP** | **rs1800625 (A > G)** |  |  |  |  |  |
| Genotype | GG / AG / AA (n) | 1 / 23 / 87 | 5 / 43 / 288^§^ | 0.117 | 0.351 |  |
|  | GG / AG / AA (freq) | 0.009 / 0.207 / 0.784 | 0.015 / 0.128 / 0.857 |  |  |  |
| Allele | G / A (n) | 25 / 197 | 53 / 619 | 0.122 | 0.366 | 1.48 (0.90–2.45) |
|  | G / A, freq | 0.113 / 0.887 | 0.079 / 0.921 |  |  |  |
| Dominant model | GG + AG / AA (freq) | 0.216 / 0.784 | 0.143 / 0.857 | 0.068 | 0.204 | 1.66 (0.96–2.86) |
| Recessive model | GG / AG + AA (freq) | 0.009 / 0.991 | 0.015 / 0.985 | 0.640 | 1.92 | 0.60 (0.07–5.21) |
|  |  |  |  |  |  |  |
| **SNP** | **rs2853807 (G > A)** |  |  |  |  |  |
| Genotype | AA / GA / GG (n) | 1 / 26 / 84 | 4 / 78 / 254^§^ | 0.968 | 2.904 |  |
|  | AA / GA / GG (freq) | 0.009 / 0.234 / 0.757 | 0.012 / 0.232 / 0.756 |  |  |  |
| Allele | A / G (n) | 28 / 194 | 86 / 586 | 0.943 | 2.829 | 0.98 (0.62–1.55) |
|  | A / G (freq) | 0.126 / 0.874 | 0.128 / 0.872 |  |  |  |
| Dominant model | AA + GA / GG (freq) | 0.243 / 0.757 | 0.244 / 0.756 | 0.986 | 2.958 | 1.00 (0.60–1.64) |
| Recessive model | AA / GA + GG (freq) | 0.009 / 0.991 | 0.012 / 0.988 | 0.801 | 2.403 | 0.75 (0.08–6.82) |

* By Chi-square test with 2×3 contingency tables for genotype; by Chi-square test with 2×2 contingency tables for allele, dominant model, and recessive model. Supposing the minor allele (m) and major allele (M), the dominant model compares mm + mM versus MM; and the recessive model compares mm versus mM + MM. If the number was less than 5, Fisher’s exact test was used instead.

^§^ n=336.

**Abbreviations:** CPFE, combined pulmonary fibrosis and emphysema; COPD, chronic obstructive pulmonary disease; SNP, single nucleotide polymorphism; n, number; freq, frequency; Pc, corrected P value; OR, odds ratio; CI, confidence interval.

**Table S2. No associations of the 3 SNPs with lung cancer in the total patients of CPFE and COPD**

|  | **Genotype/Allele** | **With lung cancer**  **n=228** | **Without lung cancer**  **n=220** | **P*** | **Pc** |
| --- | --- | --- | --- | --- | --- |
| **SNP** | **rs2070600 (C > T)** |  |  |  |  |
| Genotype* | TT / CT / CC, n | 2 / 52 / 174 | 1 / 48 / 171 | 0.828 | 2.484 |
|  | TT / CT / CC, freq | 0.009 / 0.228 / 0.763 | 0.005 / 0.218 / 0.777 |  |  |
| Allele** | T / C, n | 56 / 400 | 50 / 390 | 0.671 | 2.013 |
|  | T / C, freq | 0.123 / 0.877 | 0.114 / 0.886 |  |  |
| **SNP** | **rs1800625 (A > G)** |  |  |  |  |
| Genotype* | GG / AG / AA, n | 1 / 35 / 191^§^ | 5 / 31 / 184 | 0.231 | 0.693 |
|  | GG / AG / AA, freq | 0.004 / 0.154 / 0.841 | 0.023 / 0.141 / 0.836 |  |  |
| Allele** | G / A, n | 37 / 417 | 41 / 399 | 0.536 | 1.608 |
|  | G / A, freq | 0.081 / 0.919 | 0.093 / 0.907 |  |  |
| **SNP** | **rs2853807 (G > A)** |  |  |  |  |
| Genotype* | AA / GA / GG, n | 5 / 49 / 174 | 0 / 55 / 164^§§^ | 0.065 | 0.195 |
|  | AA / GA / GG, freq | 0.022 / 0.215 / 0.763 | 0 / 0.251 / 0.749 |  |  |
| Allele** | A / G, n | 59 / 397 | 55 / 383 | 0.864 | 2.592 |
|  | A / G, freq | 0.129 / 0.871 | 0.126 / 0.874 |  |  |

* By Chi-square test with 2×3 contingency tables. If the number was less than 5, Fisher’s exact test was used instead.

** By Chi-square test with 2×2 contingency tables.

^§^ n=227; ^§§^ n=219.

**Abbreviations:** CPFE, combined pulmonary fibrosis and emphysema; COPD, chronic obstructive pulmonary disease; SNP, single nucleotide polymorphism; n, number; freq, frequency; Pc, corrected P value.

**Table S3. Clinical characteristics of the patients with measurements of serum sRAGE levels**

| **Parameters** | **CPFE** | **COPD** | **P** |
| --- | --- | --- | --- |
| Number | 81 | 116 |  |
| Sex (female/male) | 0/81 | 0/116 |  |
| Age (years) | 72.4±6.9 | 71.8±6.9 | 0.55** |
| BMI (kg/m^2^) | 22.5±3.04 | 21.9±3.08 | 0.15** |
| Smoking history (pack-years) | 54.7±27.2 | 60.2±32.0 | 0.21** |
| Lung cancer (+/-, n)  (%) | 67/14  (82.7%) | 58/58  (50.0%) | <0.0001* |
| Pulmonary function tests |  |  |  |
| FVC (% predicted) | 98.4 (83.2-109.5) | 95.0 (80.3-112.3) | 0.62*** |
| FEV_1_ (% predicted) | 80.3 (69.6-92.8) | 67.3 (49.3-80.7) | <0.0001*** |
| FEV_1_/FVC (%) | 66.0 (59.6-73.3) | 58.6 (45.6-63.5) | <0.0001*** |
| RV (% predicted) | 117.7 (97.3-142.2) | 143.8 (125.9-165.1) | <0.0001*** |
| TLC (% predicted) | 107.3 (95.1-116.4) | 115.1 (105.3-125.8) | 0.001*** |
| DLco (% predicted) | 49.7 (39.7-60.7) | 62.5 (46.2-80.0) | <0.0001*** |
| sRAGE concentrations (pg/ml) | 558.9 (388.9-695.3) | 666.3 (429.5-931.8) | 0.014*** |
| Chest HRCT findings |  |  |  |
| LAA score | 8 (5-12) | 7 (4-13) | 0.69*** |
| Extent of interstitial change, n (%) | | | |
| Minimal  Moderate  Severe | 35 (43.2)  30 (37.0)  16 (19.8) | NF |  |
| Radiological patterns of interstitial changes, n (%) | | | |
| Honeycombing  Reticular opacity  Ground glass opacity  Traction bronchiectasis  Consolidation | 52 (64.2)  58 (71.6)  42 (51.9)  17 (21.0)  0 (0) | NF |  |
| Treatment for COPD (inhaled corticosteroids and bronchodilators) | | | |
| LAMA | 3 | 9 |  |
| LABA | 6 | 7 |  |
| ICS | 1 | 0 |  |
| LAMA+LABA | 3 | 3 |  |
| LAMA+ICS | 0 | 0 |  |
| LABA+ICS | 3 | 4 |  |
| LAMA+LABA+ICS | 1 | 2 |  |
| No pharmacotherapy | 64 | 91 |  |
| Treatment for pulmonary fibrosis (anti-fibrotic agents, systemic steroids and immunosuppressants) | | | |
| Anti-fibrotic agents |  |  |  |
| Nintedanib | 0 | NA |  |
| Pirfenidone | 1 |  |  |
| No pharmacotherapy | 80 |  |  |
| Systemic steroids | 0 |  |  |
| Immunosuppressants | 0 |  |  |

Date are expressed as mean ± standard deviation (SD) or median (interquartile range, IQR) unless otherwise stated.

*P values were analyzed by 2×2 contingency table with *, unpaired t-test with ** and Mann-Whitney U test with * * *.

**Abbreviations:** CPFE, combined pulmonary fibrosis and emphysema; COPD, chronic obstructive pulmonary disease; BMI, body mass index; FVC, forced vital capacity; FEV_1_, forced expiratory volume in 1 second; RV, residual volume; TLC, total lung capacity; DL_CO_, diffusing capacity of lung for carbon monoxide; sRAGE, soluble receptor for advanced glycation end-products; LAA, low attenuation areas; LAMA, long-acting muscarinic antagonist (Tiotropium, Glycopyrronium, Aclidinium, Umeclidinium); LABA, long-acting beta2-agonist (Indacaterol, Salmeterol, Vilanterol, Formoterol, Olodaterol); ICS, inhaled corticosteroid (Fluticasone, Budesonide, Ciclesonide, Beclometasone); NF, not found; NA, not applicable.
