# Supplementary figures and images for: The Gly82Ser mutation in AGER contributes to pathogenesis of pulmonary fibrosis in combined pulmonary fibrosis and emphysema (CPFE) in Japanese patients
Source: Sci Rep. 2020 Jul 30;10:12811. doi: 10.1038/s41598-020-69184-8 (PMC7393115; doi:10.1038/s41598-020-69184-8)

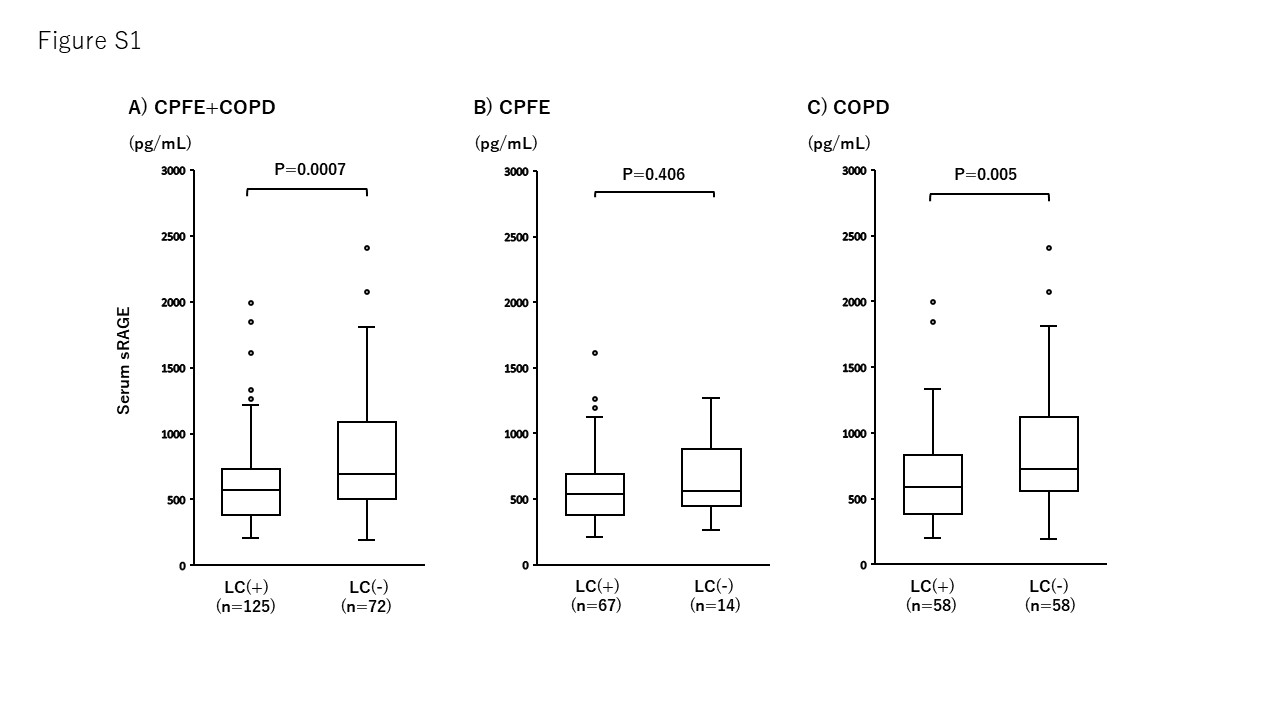

Supplement: Supplementary file 2 — Supplementary information 2. [file 41598_2020_69184_MOESM2_ESM.jpg]
